# Regional Corneal Densitometry Changes and Endothelial Stability After Cruciate-Pattern Nd:YAG Laser Posterior Capsulotomy: A Retrospective Self-Controlled Study

**DOI:** 10.3390/diagnostics16213586

**Published:** 2026-10-30

**Authors:** Yusuf Yıldırım, Elif Ertaş

**Affiliations:** 1 Department of Ophthalmology, University of Health Sciences, Gazi Yaşargil Training and Research Hospital, 21070 Diyarbakır, Türkiye; 2 Department of Biostatistics and Medical Informatics, Faculty of Medicine, Atlas University, 34408 Istanbul, Türkiye; elif.ertas@atlas.edu.tr

**Keywords:** corneal densitometry, Nd laser, posterior capsulotomy, corneal endothelium, Scheimpflug imaging

## Abstract

**Background/Objectives**: Nd:YAG laser posterior capsulotomy may be followed by subtle corneal optical changes. We evaluated regional corneal densitometry (CD) and endothelial parameters after standardized cruciate-pattern capsulotomy. **Methods**: In this retrospective, self-controlled study, 51 eyes of 51 pseudophakic patients with clinically significant posterior capsule opacification underwent capsulotomy by a single surgeon. Scheimpflug CD was assessed in the anterior, central, posterior, and full-thickness cornea across 0–2, 2–6, 6–10, and 10–12 mm zones. Best-corrected visual acuity (BCVA), intraocular pressure (IOP), pachymetry, keratometry, aberrometry, and specular microscopy were evaluated before treatment and at postoperative week 1 and month 1. **Results**: CD increased in the 0–2, 2–6, and 6–10 mm zones of the anterior and central layers, while the 10–12 mm zone showed no statistically significant time-related difference. The largest reported effect was in central-layer CD at 0–2 mm (d = 0.52). BCVA improved (decimal values, 0.32 ± 0.18 to 0.81 ± 0.13; *p* < 0.001). Endothelial cell density (ECD) and hexagonality showed no statistically significant time effects (*p* = 0.720 and *p* = 0.290); coefficient of variation decreased (*p* = 0.003), an isolated finding of uncertain clinical significance. IOP differed between week 1 and month 1 (15.0 ± 3.14 vs. 16.4 ± 2.73 mmHg) but both means were within the conventional physiological range and measurements were affected by the postoperative medication schedule. Corneal thickness, keratometric indices, and aberrations showed no significant time effects. **Conclusions**: Central and paracentral backscatter increases were detectable over one month without a statistically demonstrable deterioration in the measured endothelial or corneal structural indices. These findings do not establish endothelial equivalence or the clinical superiority of a capsulotomy pattern; controlled studies with longer follow-up are required.

## 1. Introduction

Posterior capsule opacification (PCO) is one of the most common delayed complications after cataract surgery and results from proliferation and migration of residual lens epithelial cells over the posterior capsule and intraocular lens (IOL) [1,2]. Visual acuity and visual quality may be reduced as a consequence of PCO. Neodymium:yttrium–aluminum–garnet (Nd:YAG) laser posterior capsulotomy remains the standard and highly effective treatment for visually significant PCO [2,3]. The quality of vision after capsulotomy may also be influenced by treatment-related optical defects; centrally located Nd:YAG pits, for example, may increase light scatter and reduce contrast, underscoring the importance of a precise capsulotomy technique [4].

Despite its favorable overall safety profile, Nd:YAG posterior capsulotomy is a photodisruptive procedure. Plasma formation, cavitation bubbles, acoustic microshock waves, and a local inflammatory response may produce subtle optical or structural effects in the anterior segment. Experimental studies have shown that corneal endothelial injury depends not only on laser energy but also on the distance between the laser focus and the endothelium [5,6].

These effects are clinically relevant because corneal transparency is a major determinant of optical quality, and even low-level increases in corneal light scatter may influence contrast sensitivity and glare when they involve the central optical axis. Scheimpflug-based corneal densitometry (CD) provides an objective method for quantifying backscattered light in grayscale units (GSU; 0 = maximum transparency and 100 = complete opacity) across a 12-mm corneal diameter and permits both zonal and depth-resolved analysis. Normative data demonstrate the lowest densitometry values centrally, with increasing values toward the periphery. However, brightness artifacts can affect densitometry estimates, with a greater influence reported in the peripheral cornea and stromal layers beyond approximately 7.5 mm [7].

Corneal densitometry can identify subtle or subclinical optical changes even when the cornea appears clear on routine slit-lamp examination. Objective CD changes have been demonstrated after chalazion excision and during follow-up after laser peripheral iridotomy for acute primary angle closure, even after clinically visible corneal edema had resolved [8,9]. These observations support CD as a sensitive outcome measure for low-grade corneal optical alterations that may not be captured by pachymetry or biomicroscopy alone.

In our previous Nd posterior capsulotomy cohort, CD increased in the 6–10 mm annulus across the anterior, central, and posterior layers, whereas the 0–2 and 2–6 mm zones showed no significant change [10]. That investigation did not include specular-microscopy endpoints and assessed postoperative outcomes only at month 1. Moreover, measurable anterior-segment changes have been reported as early as postoperative week 1 after Nd posterior capsulotomy, supporting evaluation at both week 1 and month 1 [11].

Previous studies have compared cruciate and circular Nd posterior capsulotomy techniques with respect to visual outcomes, intraocular pressure, laser energy requirements, and optical quality. Kara et al. reported greater laser energy requirements and a transient increase in macular thickness following circular capsulotomy compared with the cruciate technique [12]. Kim et al. found no significant difference in endothelial cell count changes between cruciate and modified circular capsulotomy techniques [13]. Furthermore, Li et al. demonstrated lower intraocular light scattering following circular capsulotomy compared with the cruciate pattern [14]. However, these investigations did not assess regional and layer-specific Scheimpflug-based corneal densitometry, and the relationship between capsulotomy geometry and corneal backscatter remains insufficiently characterized.

To further investigate corneal changes after Nd posterior capsulotomy, the present retrospective, self-controlled study evaluated a separate cohort of 51 eyes treated with a standardized cruciate (+) capsulotomy pattern centered on the visual axis. We assessed the spatial distribution of CD changes across central, paracentral, and peripheral corneal zones and determined whether these changes were accompanied by endothelial alterations on specular microscopy at postoperative week 1 and month 1. By integrating regional and layer-specific CD measurements with endothelial assessments at multiple postoperative time points, this study aimed to provide a more comprehensive characterization of short-term corneal responses following cruciate-pattern Nd posterior capsulotomy.

## 2. Materials and Methods

### 2.1. Study Design, Setting, and Ethics

This retrospective, observational, self-controlled study was conducted at the Department of Ophthalmology of a tertiary referral hospital. Medical records dating from 1 January 2021 to 1 August 2026 were retrospectively reviewed to identify patients who had undergone Nd laser posterior capsulotomy and completed their clinical follow-up within this period. The study was approved by the hospital Clinical Research Ethics Committee (Approval No. 364; 14 August 2026) and was conducted in accordance with the Declaration of Helsinki. Written informed consent was obtained from all participants. Preoperative measurements were compared with postoperative week-1 and month-1 measurements, with each eye serving as its own control.

### 2.2. A Priori Sample Size Calculation

An a priori sample size calculation was performed during the study planning phase to determine the sample size required to detect clinically meaningful longitudinal changes in corneal densitometry. The calculation was based on a one-way repeated-measures analysis of variance (ANOVA) with three assessment time points (preoperative, postoperative week 1, and postoperative month 1). A medium standardized effect size (Cohen’s f = 0.25) was assumed for statistical planning, consistent with conventional effect size classifications [15,16]. A Type I error rate of 5% (α = 0.05) and statistical power of 90% (1 − β = 0.90) were specified. The final study population comprised 51 patients (51 eyes).

### 2.3. Patient Selection: Inclusion and Exclusion Criteria

**Figure 1:**
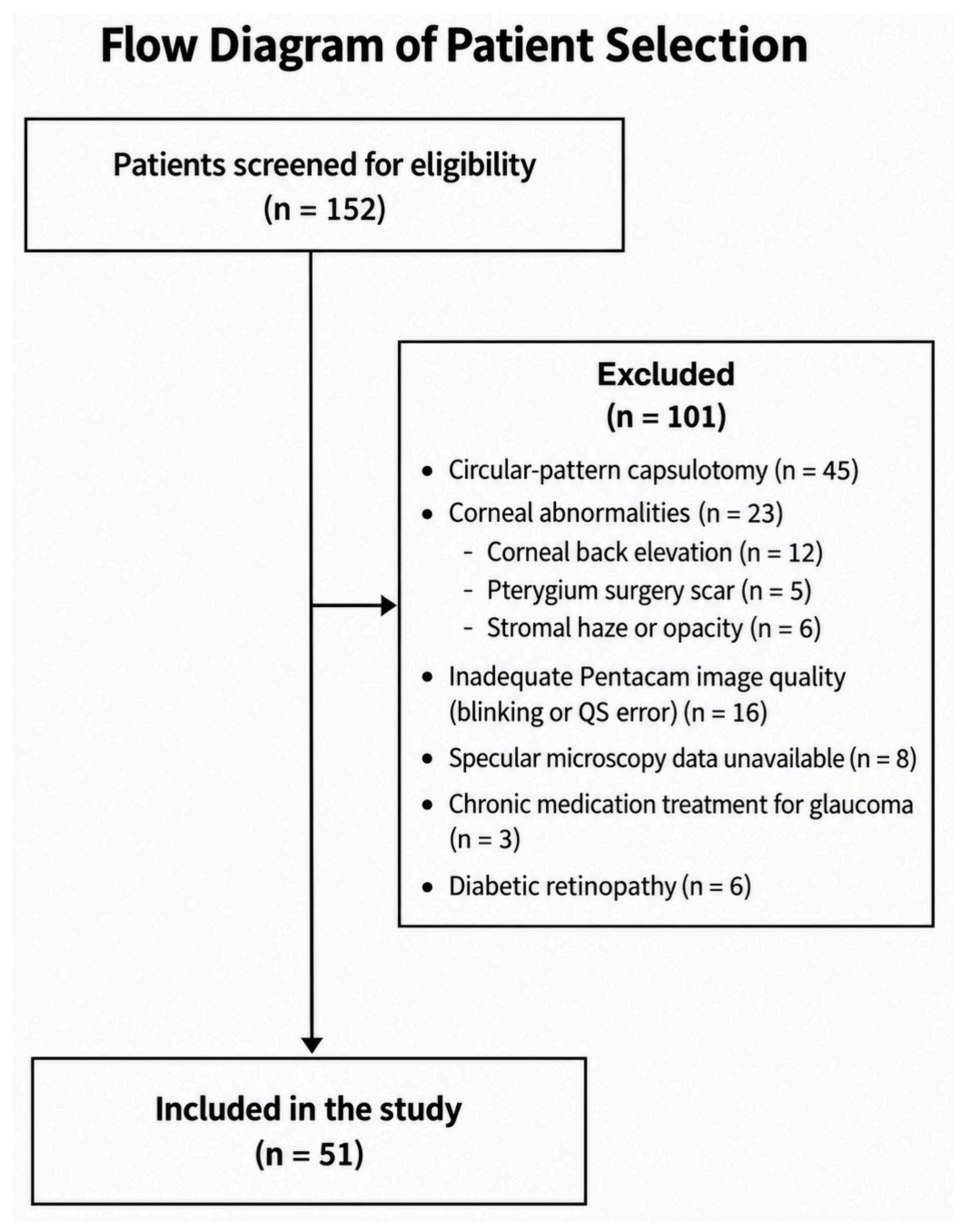
Flow diagram of patient selection. Among 152 patients screened for eligibility, 101 were excluded because of circular-pattern capsulotomy (*n* = 45), corneal abnormalities (*n* = 23), inadequate Pentacam image quality (*n* = 16), unavailable specular microscopy data (*n* = 8), chronic antiglaucomatous medication use (*n* = 3), or diabetic retinopathy (*n* = 6). The final study population comprised 51 eyes of 51 patients who underwent cruciate-pattern Nd:YAG laser posterior capsulotomy.

Consecutive pseudophakic patients who underwent Nd:YAG laser posterior capsulotomy for clinically significant posterior capsule opacification (PCO) were retrospectively screened. Eligibility required PCO associated with a loss of at least two Snellen lines relative to the best-corrected visual acuity achieved after cataract surgery, technically acceptable Pentacam and specular microscopy examinations, and complete measurements at baseline, postoperative week 1, and postoperative month 1. Exclusion criteria included corneal scarring, dystrophy, ectasia, or clinically significant ocular surface disease; glaucoma or ocular hypertension; previous uveitis or intraocular inflammation; retinal disease; ocular trauma; previous ocular surgery other than uncomplicated cataract surgery; contact lens wear; relevant medication use or systemic disease; and missing or technically inadequate measurements [10]. A total of 152 patients were screened for eligibility. Of these, 101 patients were excluded: 45 underwent circular-pattern rather than cruciate-pattern capsulotomy; 23 had corneal abnormalities, including abnormal posterior corneal elevation *(n* = 12), corneal scarring following pterygium surgery (*n* = 5), and stromal haze or opacity (*n* = 6); 16 had inadequate Pentacam image quality because of blinking artifacts or quality specification (QS) errors; 8 had unavailable specular microscopy data; 3 received chronic antiglaucomatous medication; and 6 had diabetic retinopathy. Consequently, 51 eyes of 51 patients fulfilled the eligibility criteria and were included in the final analysis (Figure 1). All 51 patients included in the final analysis had complete preoperative, postoperative week-1, and postoperative month-1 follow-up data. Among the 152 patients whose records were reviewed, no patient was excluded specifically because of loss to follow-up or unavailable follow-up examination records.

### 2.4. Ophthalmic Examination

All patients were examined before the procedure and at postoperative week 1 and month 1. Best-corrected visual acuity (BCVA) was initially recorded using Snellen notation and subsequently converted to decimal visual acuity for analysis. The decimal equivalent was calculated by dividing the Snellen numerator by the denominator (e.g., 20/20 = 1.0, 20/40 = 0.5, and 20/200 = 0.1), according to the Snellen-to-decimal conversion shown in Table 1 [10]. No logMAR transformation was performed. The decimal BCVA values were used directly for all statistical analyses. Refraction was measured using an automated refractometer (RM-KR8800; Topcon Corp., Tokyo, Japan), and intraocular pressure (IOP) was measured using noncontact tonometry (TX-20P; Canon Inc., Tokyo, Japan).

### 2.5. Scheimpflug Imaging and Corneal Densitometry

Corneal tomography and CD measurements were obtained using a rotating Scheimpflug camera (Pentacam HR; Oculus GmbH, Wetzlar, Germany). All measurements were performed by the same experienced operator in the same dim examination room under comparable environmental conditions. Three consecutive measurements were obtained for each eye; only scans marked “OK” by the device and meeting quality criteria were included, and the mean of the three valid measurements was used for analysis [10,17].

**Figure 2:**
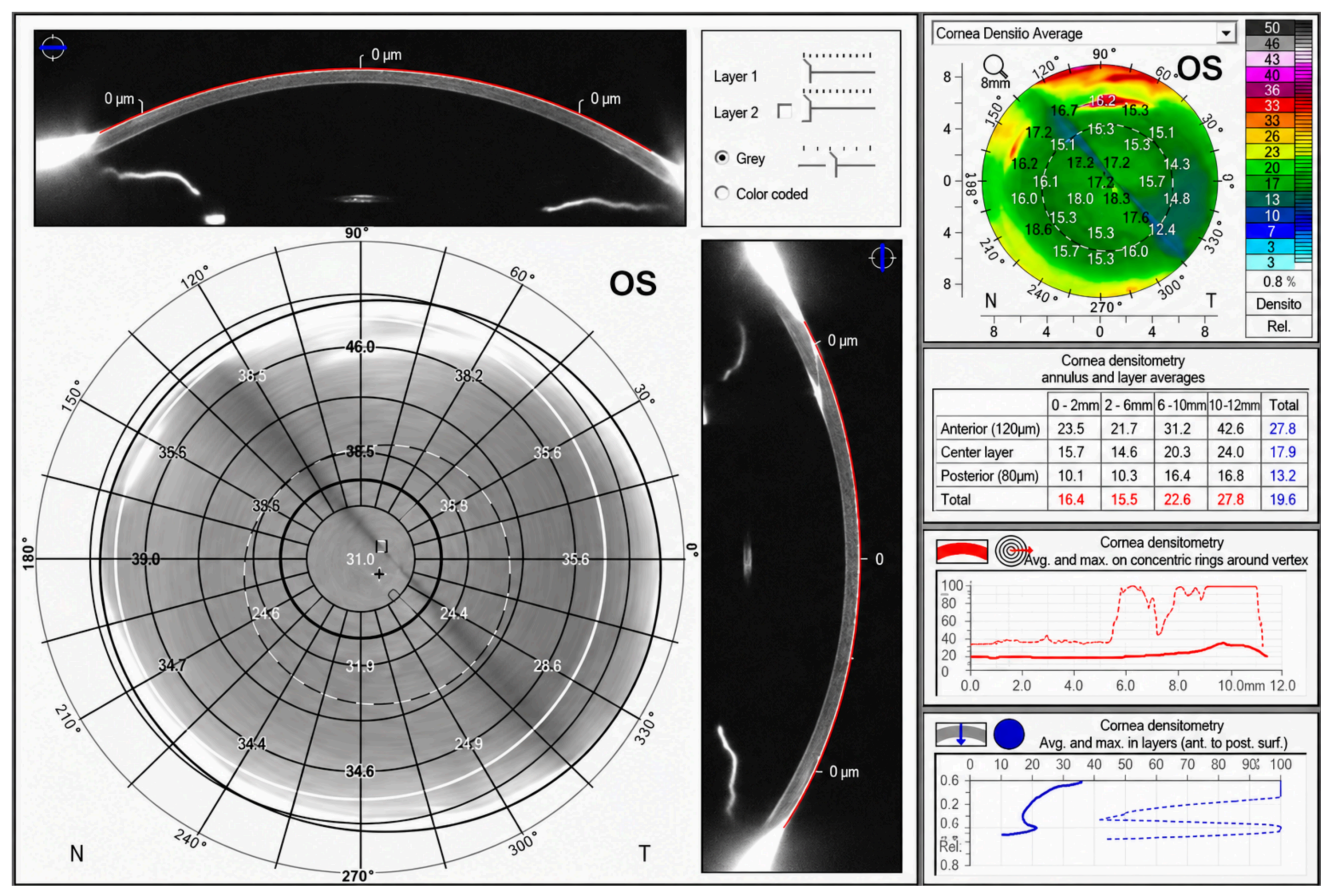
Representative Scheimpflug corneal densitometry analysis. Corneal densitometry values are shown across concentric annular zones and corneal layers, including the anterior, central, and posterior layers. Measurements are expressed in grayscale units.

Corneal density was automatically measured over a 12-mm corneal diameter and analyzed in four concentric zones: 0–2, 2–6, 6–10, and 10–12 mm. The anterior layer was defined as the first 120 μm from the anterior corneal surface, the posterior layer as the final 60 μm adjacent to the posterior surface, and the central layer as the tissue between these two layers. For each corneal layer and for the full thickness, zonal CD values and an overall CD value were recorded (Figure 2) [10,17].

**Figure 3:**
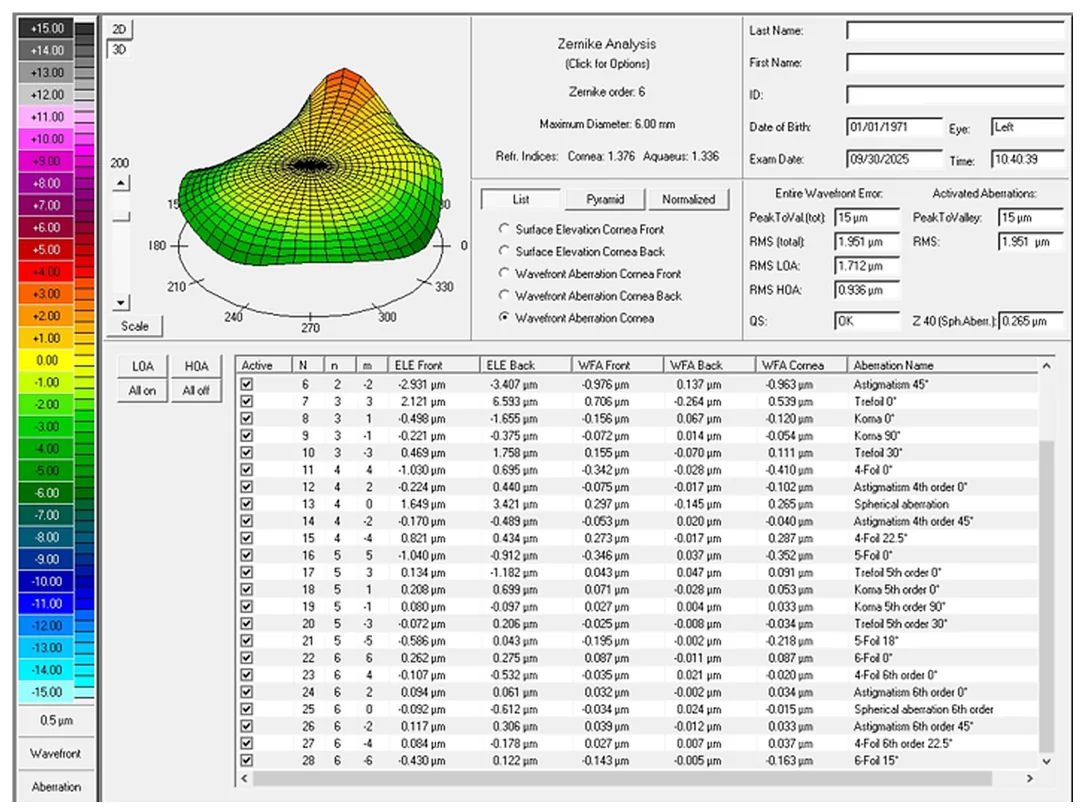
Representative corneal wavefront aberration output obtained using Scheimpflug-based imaging. Quantitative parameters, including total root mean square, higher-order aberrations, spherical aberration, and coma values, are displayed.

Flat keratometry (K1), steep keratometry (K2), maximum keratometry (Kmax), and central corneal thickness (CCT) were recorded. Aberrometric analysis included total root mean square (RMS), higher-order aberration RMS (RMS HOA), spherical aberration, and coma components at 0° and 90°, calculated by the Pentacam software(version: 1.36r03) over the central 6-mm optical zone (Figure 3).

### 2.6. Specular Microscopy and Endothelial Parameters

Corneal endothelial evaluation was performed using noncontact specular microscopy (SP-3000P; Topcon Corporation, Tokyo, Japan). All measurements were obtained from the central cornea using a standardized acquisition protocol, with patients instructed to maintain steady fixation during image capture. Images with adequate focus and clearly identifiable endothelial cell borders were selected for analysis, whereas poor-quality or decentered images were excluded. Automated endothelial cell analysis was reviewed to ensure appropriate cell-border identification before measurements were recorded. Endothelial cell density (ECD; cells/mm^2^), coefficient of variation of endothelial cell area (CV; %, a measure of polymegathism), and percentage of hexagonal cells (HEX; a measure of pleomorphism) were recorded preoperatively and at postoperative week 1 and month 1.

### 2.7. Nd:YAG Laser Posterior Capsulotomy

**Figure 4:**
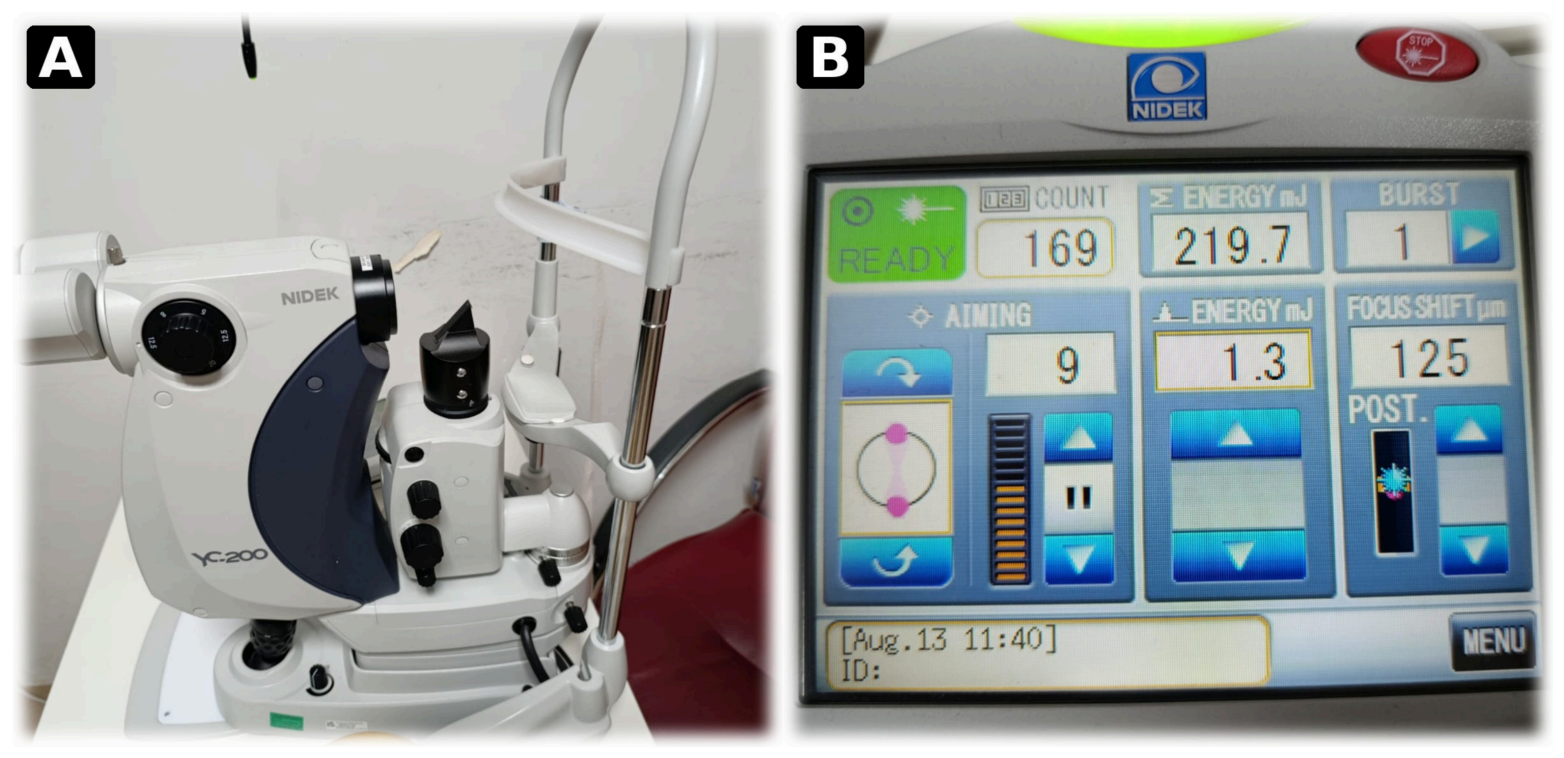
(**A**) YC-200 Ophthalmic YAG Laser System (NIDEK Co., Ltd., Gamagori, Aichi, Japan). (**B**) Laser control panel showing a pulse energy of 1.3 mJ, a pulse count of 169, and a total energy of 219.7 mJ. YAG, yttrium–aluminum–garnet.

To minimize operator-related variability, all Nd:YAG posterior capsulotomy procedures were performed by the same experienced surgeon (Y.Y.) using the YC-200 Ophthalmic YAG Laser System (NIDEK Co., Ltd., Gamagori, Aichi, Japan) and a capsulotomy contact lens. Initial pulse energy was set at 1.0 mJ and increased gradually when required according to posterior capsule density. Pulse count and total laser energy were recorded for each procedure (Figure 4).

### 2.8. Cruciate (+) Capsulotomy Technique

**Figure 5:**
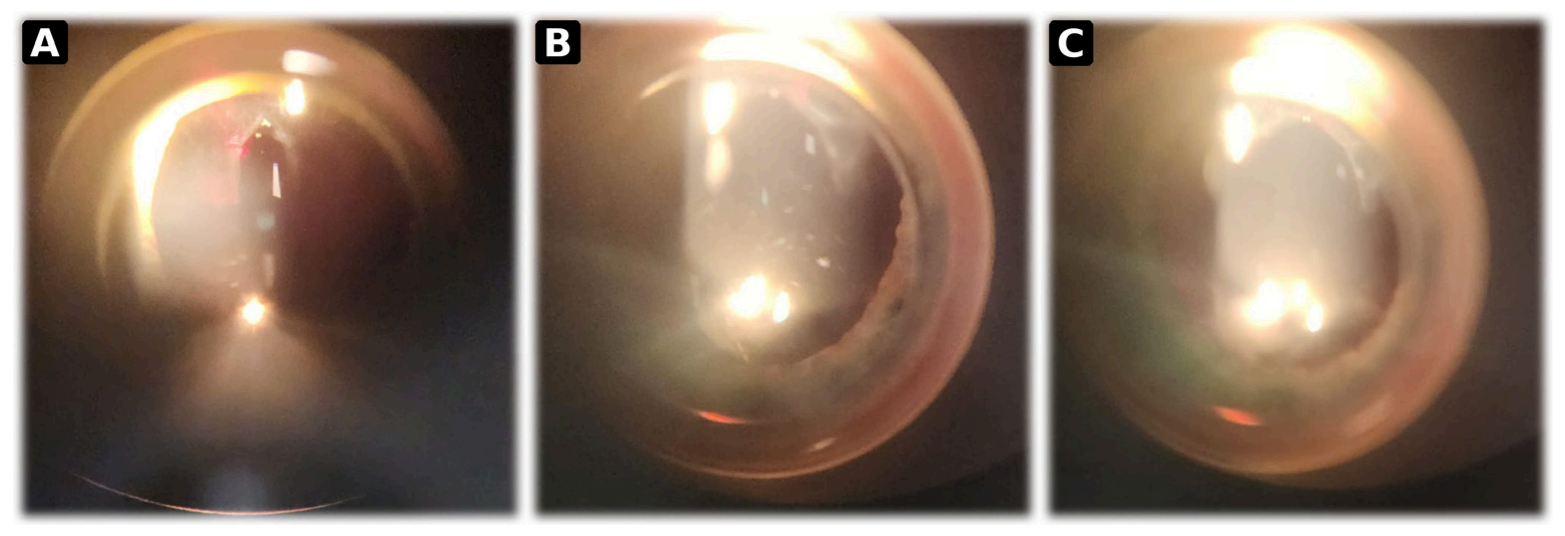
Demonstration of the cruciate-pattern Nd:YAG laser capsulotomy technique: (**A**) creation of the vertical arm, (**B**) creation of the horizontal arm, and (**C**) completed capsulotomy opening. Nd:YAG, neodymium:yttrium–aluminum–garnet.

**Figure 6:**
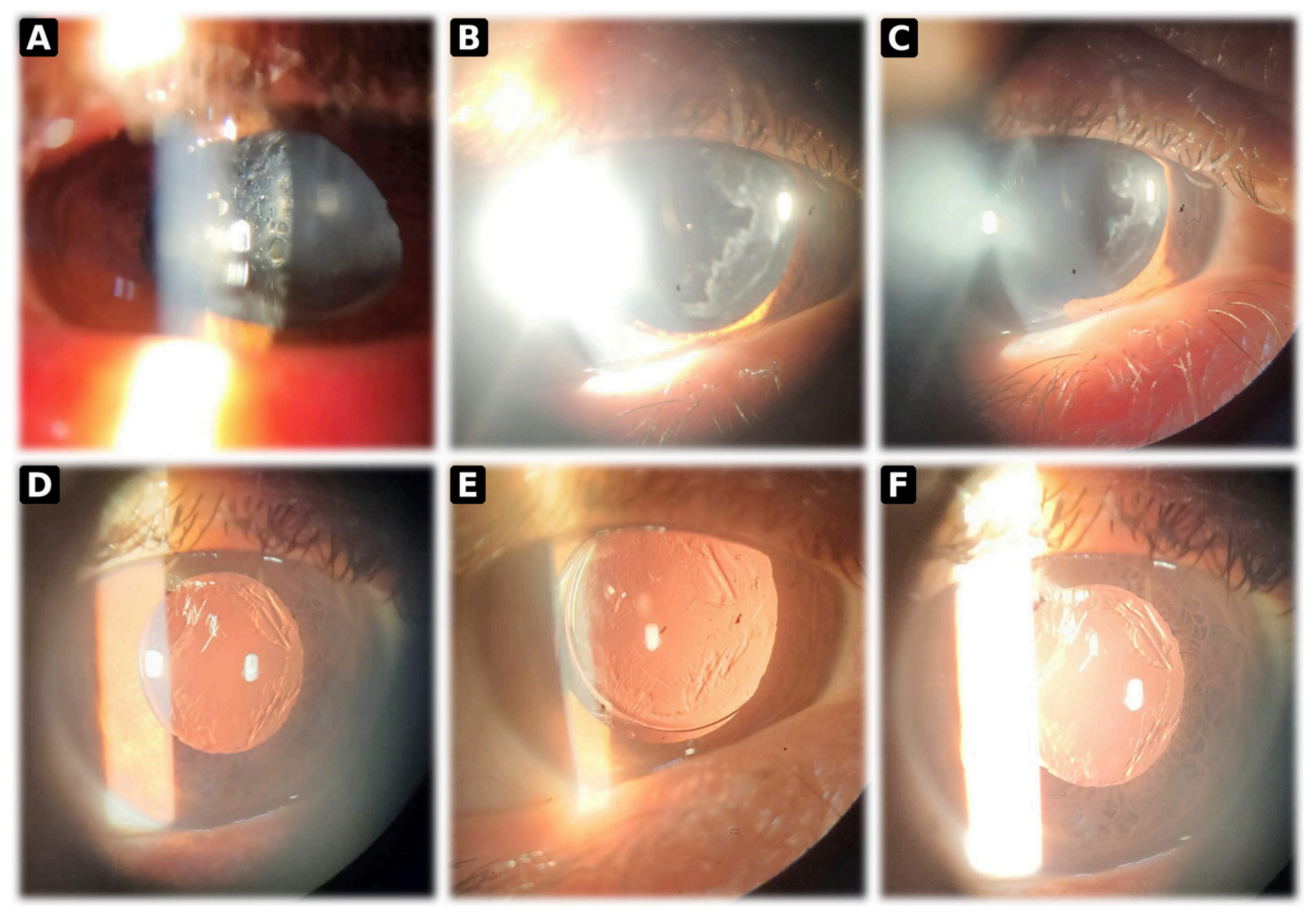
(**A**) Posterior capsule opacification. (**B**,**C**) Creation of the cruciate-pattern capsulotomy. (**D**–**F**) Appearance of the completed capsulotomy.

After adequate pupillary dilation and appropriate focusing through the capsulotomy lens, the posterior capsule was opened in a cruciate (+) pattern centered on the visual axis. The arms of the cruciate opening were enlarged symmetrically while avoiding IOL pitting, creating an adequate central optical opening. Capsulotomy diameter was recorded after completion of the procedure (Figure 5 and Figure 6).

### 2.9. Postoperative Treatment

Postoperatively, all patients received 1% prednisolone acetate (Pred Forte; Allergan, Irvine, CA, USA) six times daily, 0.5% apraclonidine hydrochloride (Iopidine; Alcon Laboratories, Fort Worth, TX, USA) twice daily, and fixed-combination 0.5% dorzolamide/2% timolol maleate (Tomec; Abdi İbrahim Pharmaceuticals, Istanbul, Türkiye) twice daily for 1 week. After the week-1 visit, apraclonidine and dorzolamide/timolol were discontinued, and prednisolone acetate was gradually tapered.

### 2.10. Statistical Analysis

Statistical analyses were performed using IBM SPSS Statistics version 25.0 (IBM Corp., Armonk, NY, USA). Continuous variables were expressed as mean ± standard deviation (SD) or median (interquartile range [IQR]), according to their distributional characteristics. Normality was assessed using the Kolmogorov–Smirnov test.

Longitudinal comparisons across three time points (preoperative, postoperative week 1, and postoperative month 1) were performed using repeated-measures analysis of variance (ANOVA) for variables meeting the relevant parametric assumptions and the Friedman test for variables analyzed nonparametrically. Repeated-measures ANOVA was applied to all corneal densitometry parameters, best-corrected visual acuity, intraocular pressure, central corneal thickness, keratometric parameters (K1, K2, and Kmax), endothelial cell-area coefficient of variation, hexagonality, total root mean square (RMS), higher-order aberration RMS, and spherical aberration. The Friedman test was used for endothelial cell density (ECD), coma 0°, and coma 90°.

When a statistically significant overall time effect was detected, post-hoc pairwise comparisons were performed with Bonferroni correction to control the family-wise Type I error rate. Cohen’s d effect sizes were reported for parametric comparisons.

Associations between total Nd laser energy and changes in corneal densitometry parameters over three intervals (baseline to week 1, baseline to month 1, and week 1 to month 1) were assessed using Pearson correlation analysis. These correlation analyses were considered exploratory, and no adjustment for multiple testing was applied. Therefore, isolated statistically significant correlations were interpreted cautiously.

All statistical tests were two-sided, and *p* < 0.05 was considered statistically significant.

## 3. Results

Fifty-one eyes of 51 patients undergoing cruciate-pattern Nd:YAG laser posterior capsulotomy were included. Mean age was 67.1 ± 8.1 years, 29 patients (56.9%) were women, and 26 treated eyes (51.0%) were left eyes. All patients had a foldable IOL. Mean refraction was −0.90 ± 1.86 *D*. The mean interval between cataract surgery and laser capsulotomy was 1133.02 ± 1473.98 days. Mean pulse energy was 1.02 ± 0.05 mJ, mean pulse count was 120.7 ± 68.8, mean total energy was 119.22 ± 68.13 mJ, and mean capsulotomy diameter was 4.39 ± 0.51 mm (Table 2).

**Figure 7:**
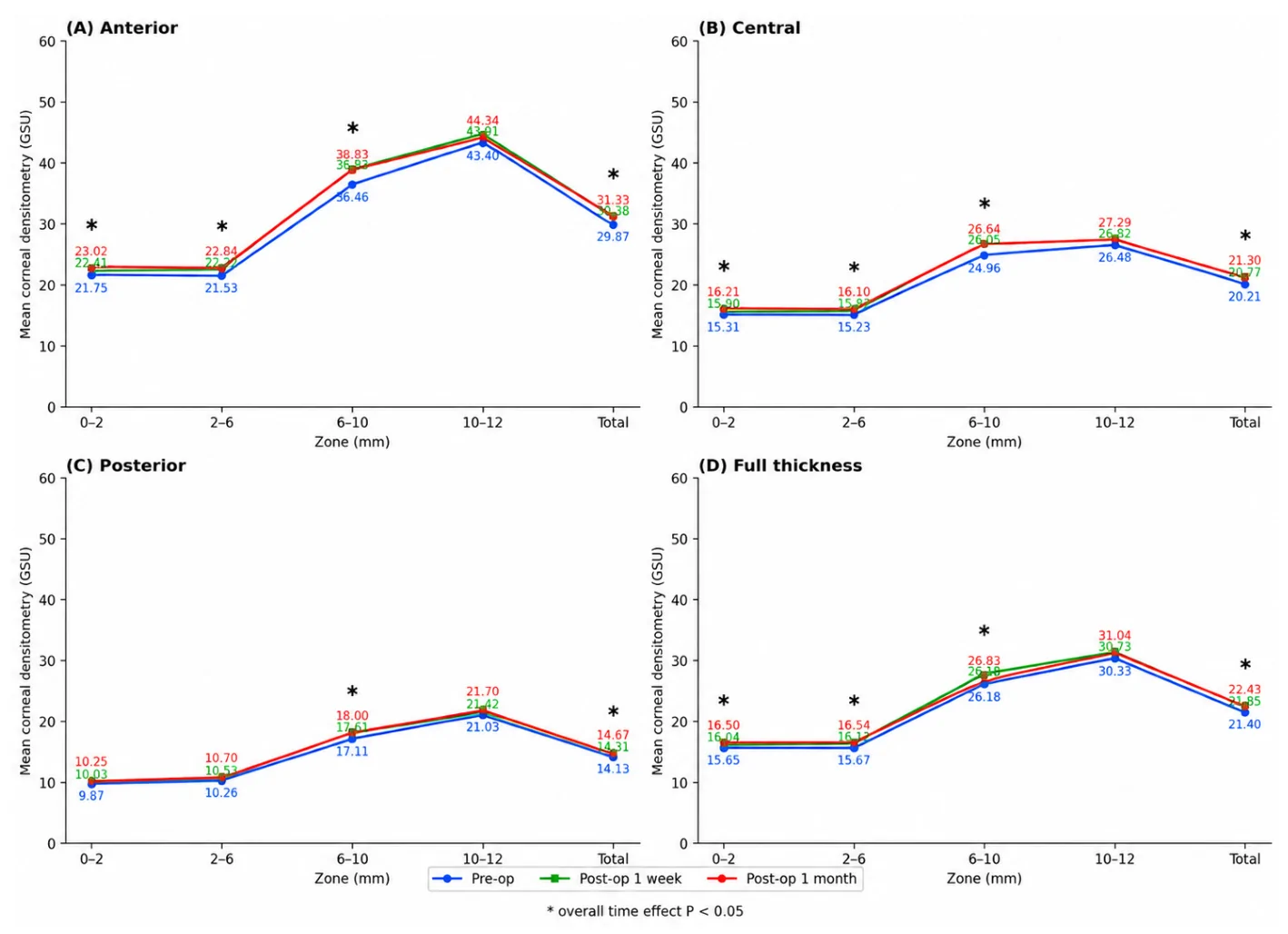
Corneal densitometry changes after cruciate-pattern Nd:YAG laser posterior capsulotomy in the anterior (**A**), central (**B**), posterior (**C**), and full-thickness (**D**) corneal layers. Values are shown for the preoperative, postoperative week 1, and month 1 examinations. *p* < 0.05 indicates a significant time effect. CD, corneal densitometry; GSU, grayscale units; Nd:YAG, neodymium:yttrium–aluminum–garnet.

Across the preoperative, week-1, and month-1 assessments, CD changes varied by layer and zone. In the anterior layer, significant time effects were observed in the 0–2, 2–6, and 6–10 mm zones and in the overall value (*p* < 0.001, *p* < 0.001, *p* < 0.001, and *p* = 0.001, respectively), with preoperative-to-month-1 effect sizes of d = 0.46, 0.36, and 0.20 for the respective zones; the 10–12 mm zone remained stable (*p* = 0.540). Similar changes occurred in the central layer in the 0–2, 2–6, and 6–10 mm zones and in the overall value (all *p* < 0.001), with corresponding effect sizes of d = 0.52, 0.42, 0.25, and 0.30; the 10–12 mm zone remained unchanged (*p* = 0.210). Posterior-layer changes were limited to the 6–10 mm zone (*p* = 0.007; d = 0.24) and the overall value (*p* = 0.040). Full-thickness CD showed significant time effects in the 0–2, 2–6, and 6–10 mm zones and in the overall value (*p* < 0.001, *p* < 0.001, *p* < 0.001, and *p* = 0.001, respectively), with effect sizes of d = 0.49, 0.38, 0.09, and 0.25; the 10–12 mm zone remained stable (*p* = 0.360) (Table 3; Figure 7).

**Figure 8:**
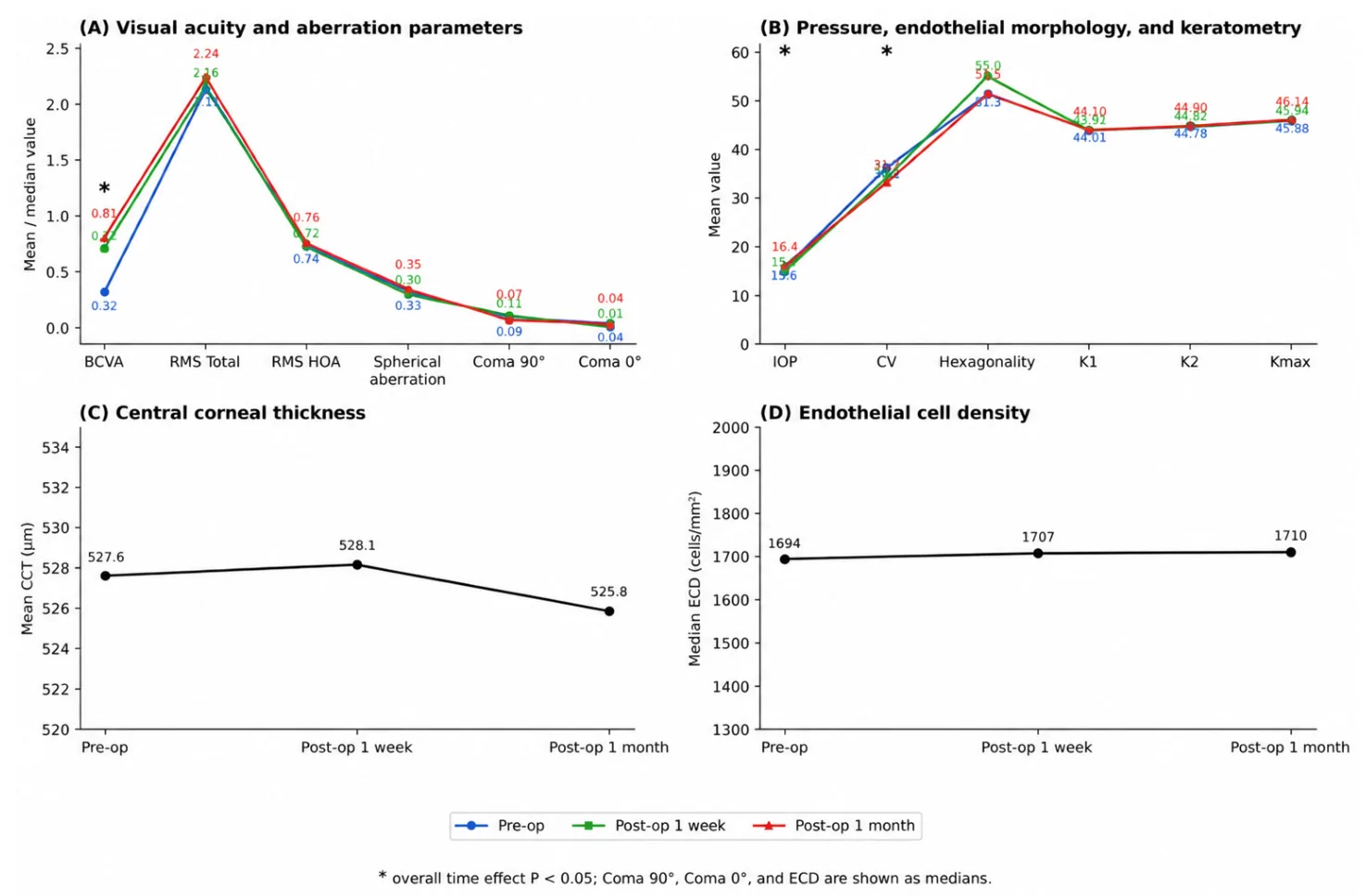
Changes in visual, aberrometric, endothelial, keratometric, pachymetric, and intraocular pressure parameters after cruciate-pattern Nd:YAG laser posterior capsulotomy. Measurements are shown preoperatively and at postoperative week 1 and month 1. *p* < 0.05 indicates a significant time effect. BCVA, best-corrected visual acuity; CCT, central corneal thickness; CV, coefficient of variation; ECD, endothelial cell density; HOA, higher-order aberrations; IOP, intraocular pressure; K1, flat keratometry; K2, steep keratometry; Kmax, maximum keratometry; RMS, root mean square; Nd:YAG, neodymium:yttrium–aluminum–garnet.

Coefficient of variation decreased significantly over time (*p* = 0.003), with a preoperative-to-month-1 effect size of d = 0.46. In contrast, neither hexagonality (*p* = 0.290) nor ECD (*p* = 0.720) showed a statistically significant time effect. To further characterize ECD changes, paired median differences with 95% confidence intervals were calculated. The median ECD change was +4.2 cells/mm^2^ from baseline to week 1 (95% CI, −63.0 to +47.4), −18.5 cells/mm^2^ from baseline to month 1 (95% CI, −58.4 to +16.9), and +0.6 cells/mm^2^ from week 1 to month 1 (95% CI, −44.3 to +63.2). All three confidence intervals included zero. These nonsignificant findings do not establish endothelial equivalence or exclude smaller clinically relevant changes (Table 4; Figure 8).

Best-corrected visual acuity improved significantly from 0.32 ± 0.18 preoperatively to 0.71 ± 0.15 at week 1 and 0.81 ± 0.13 at month 1 (*p* < 0.001), with all pairwise comparisons significant (Table 4).

IOP showed an overall time effect (*p* = 0.004), with a reported week-1 versus month-1 difference (15.0 ± 3.14 vs. 16.4 ± 2.73 mmHg). Both group means fell within a conventional physiological range; individual IOP elevations and their clinical importance cannot be established from these summary statistics. The week-1 examination occurred during prophylactic IOP-lowering therapy (Table 4; Figure 8).

Central corneal thickness and keratometric parameters (K1, K2, and Kmax) remained stable throughout follow-up (all *p* > 0.05) (Table 4; Figure 8).

Aberrometric parameters remained stable throughout follow-up, including total RMS (*p* = 0.510), RMS HOA (*p* = 0.360), spherical aberration (*p* = 0.060), coma 90° (*p* = 0.940), and coma 0° (*p* = 0.360) (Table 4).

Weak positive correlations were observed between total Nd:YAG laser energy and changes in corneal densitometry in the 6–10 mm annulus from baseline to postoperative week 1 in the anterior layer (r = 0.316, *p* = 0.024), posterior layer (r = 0.295, *p* = 0.035), and full-thickness measurements (r = 0.292, *p* = 0.037). The correlation in the central layer of the 6–10 mm annulus did not reach statistical significance (r = 0.255, *p* = 0.071). No statistically significant correlations were identified in the remaining analyses, including baseline-to-month 1 and week 1-to-month 1 comparisons (all *p* ≥ 0.05).

Overall, these findings suggest that higher total laser energy may be associated with greater early (first-week) changes in corneal densitometry within the 6–10 mm annulus. However, the observed correlations were weak and limited to the anterior, posterior, and full-thickness measurements, whereas the correlation in the central layer was not statistically significant (Table 5).

## 4. Discussion

In this retrospective, self-controlled study, CD changes after cruciate-pattern Nd:YAG posterior capsulotomy were characterized by significant increases from preoperative values to month 1 in the 0–2, 2–6, and 6–10 mm zones of the anterior and central layers, while the 10–12 mm peripheral zone remained stable across all time points in all layers. Posterior-layer changes were restricted to the 6–10 mm zone and the posterior overall value. The largest standardized effect was observed in the central-layer 0–2 mm zone between the preoperative and month-1 examinations and was moderate in magnitude (d = 0.52). Because most other effect sizes were small, the observed pattern is more consistent with an anatomically localized, centrally weighted change in corneal backscatter than with diffuse corneal deterioration.

The distribution of changes in CD differed markedly from that observed in our previous series. In that study, CD values remained stable across all corneal layers in the 0–2, 2–6, and 10–12 mm zones, whereas a significant increase was observed in all corneal layers only within the 6–10 mm zone [10]. Central corneal thickness, keratometric, and aberrometric parameters also remained stable. In the present cruciate-pattern cohort, however, CD increases were concentrated in the central and paracentral 0–2 and 2–6 mm zones, which correspond closely to the corneal pathway of the laser beam, with the largest effect in the central 0–2 mm zone. This spatial concordance supports the hypothesis that capsulotomy geometry may influence the distribution of the densitometric response. Nevertheless, because circular and cruciate capsulotomy patterns were not directly compared in parallel groups and other between-cohort differences cannot be excluded, the association does not establish causality.

Experimental evidence provides possible, rather than demonstrated, explanations for post-capsulotomy changes in corneal backscatter; photodisruption-related plasma effects, acoustic waves, inflammation, and focus-to-endothelium distance may be relevant [5,6]. These mechanisms were not tested in the present observational cohort. Previous reports described ECD reductions following Nd:YAG capsulotomy [2,18,19,20]; however, the absence of a significant ECD change in our 51-eye cohort does not exclude a clinically relevant small loss. To further quantify the uncertainty surrounding ECD changes, paired median differences with 95% confidence intervals were estimated using 50,000 bootstrap resamples. The median ECD changes were +4.2 cells/mm^2^ from baseline to week 1 (95% CI, −63.0 to +47.4), −18.5 cells/mm^2^ from baseline to month 1 (95% CI, −58.4 to +16.9), and +0.6 cells/mm^2^ from week 1 to month 1 (95% CI, −44.3 to +63.2). Because all confidence intervals included zero, these findings do not demonstrate a consistent directional change in ECD; however, smaller clinically relevant endothelial changes cannot be excluded.

Chen et al. described endothelial changes related to the interval between cataract surgery and Nd:YAG capsulotomy [21]. The median cataract-to-capsulotomy interval in the present sample was 740 days. However, because neither interval-stratified outcomes nor an untreated comparator were examined, our findings cannot establish that a longer interval protects the endothelium.

The decrease in endothelial cell-area CV from 36.4% preoperatively to 33.3% at month 1 represents a change toward lower polymegathism. Nevertheless, because ECD and hexagonality showed no statistically significant time effects, we did not interpret this isolated finding as endothelial improvement. Cellular rearrangement, differences in the sampled endothelial area, and measurement variability are possible explanations. Small changes in specular-microscopy indices should therefore be interpreted cautiously, particularly when other endothelial measures remain unchanged. Longitudinal laser studies likewise emphasize contextual interpretation of endothelial parameters over time [22]. The median baseline ECD of 1694 cells/mm^2^ should also be considered when generalizing short-term endothelial stability to eyes with very low endothelial reserve or to longer follow-up periods.

Central corneal thickness and keratometric parameters remained stable, consistent with previous Nd:YAG capsulotomy studies [2,3,11,18,19]. Studies of panretinal photocoagulation have likewise not shown major deterioration in CCT or endothelial parameters [23,24]. The absence of accompanying CCT or keratometric change suggests that the densitometric backscatter response may be a more sensitive optical variable than gross changes in corneal thickness or shape.

At the available postoperative time points, mean IOP did not indicate a sustained clinically substantial increase, consistent with previous reports [2,11,18,19,25]. Nevertheless, immediate postlaser pressure spikes were not assessed, and week-1 IOP was measured during apraclonidine and dorzolamide/timolol treatment. The numerical week-1-to-month-1 difference may therefore reflect treatment timing as well as other factors rather than a direct effect of the laser.

Scheimpflug densitometry quantifies corneal backscatter at both layer and zonal levels and may reveal changes that are not obvious on biomicroscopy. In several clinical models, CD alterations have been observed without parallel changes in CCT or keratometry [8,26,27,28].

Nevertheless, Scheimpflug-based CD is not completely independent of optical artifact. Arizcuren et al. reported a positive correlation between overall brightness artifact and CD and found a greater artifact effect in the peripheral cornea beyond approximately 7.5 mm [7]. Because that work used the Galilei system, its quantitative thresholds cannot be transferred directly to Pentacam measurements. In the present study, CD remained stable in the 10–12 mm peripheral zone in the anterior, central, and posterior layers, whereas the largest effect occurred in the central layer of the 0–2 mm zone. This distribution makes a predominantly peripheral brightness artifact an unlikely explanation for the observed pattern. However, other optical factors, including PCO/anterior capsule opacification, capsulorhexis and IOL optic-edge reflections, eye alignment, and tear-film quality, were not quantified; therefore, an artifact contribution cannot be completely excluded [29].

The clinical significance of the densitometric changes should be interpreted together with the functional and structural outcomes. Best-corrected visual acuity improved markedly after Nd capsulotomy, consistent with previous studies reporting significant visual improvement following treatment [2,3,19,20,21,25]. Despite the observed small-to-moderate increases in CD, CCT, keratometric and aberrometric parameters showed no statistically significant changes; ECD and hexagonality likewise showed no statistically significant time effects [2,3,11,18,20,21]. In our previous study involving eyes treated with a cruciate-pattern capsulotomy, we found no significant association between total laser energy and densitometric changes in any corneal layer. In the present series of 51 eyes undergoing cruciate-pattern Nd posterior capsulotomy, however, significant increases in corneal densitometry were observed at postoperative week 1 in the anterior, posterior, and full-thickness measurements within the 6–10 mm annulus compared with preoperative values. Although the 6–10 mm densitometric increases remained detectable at month 1, the weak correlations between total laser energy and CD change were confined to the baseline-to-week-1 interval and were not observed for baseline-to-month-1 changes [10]. These findings suggest that the densitometric changes were not associated with measurable corneal structural deterioration or loss of high-contrast visual acuity during the 1-month follow-up. However, visual acuity and corneal wavefront measurements may not fully reflect glare, halo, or intraocular straylight; future studies should therefore investigate whether central CD changes are associated with objective straylight and contrast-sensitivity measures [4].

The main strengths of this study include the within-eye design, evaluation at three time points, and analysis of CD according to both corneal layer and concentric zone. Combining Scheimpflug imaging with specular microscopy, keratometry, pachymetry, and aberrometry provided an integrated assessment of optical and structural corneal changes. In addition, averaging three quality-approved Scheimpflug acquisitions for each eye reduced measurement variability, while reporting effect sizes allowed statistically significant changes to be interpreted according to their magnitude.

Several limitations warrant emphasis. This retrospective, single-center before–after study included only 51 complete cases and lacked an untreated parallel control group (such as eligible fellow eyes or comparable pseudophakic eyes). Thus, secular effects, measurement drift, effects associated with the time since cataract surgery, and other temporal confounders cannot be separated definitively from postlaser changes. The retrospective design and inclusion of patients with complete follow-up may also introduce selection bias, although a patient-selection flow diagram was added to document screening and exclusion reasons. The one-month follow-up limits conclusions regarding long-term corneal transparency and endothelial safety. In addition, all procedures used a cruciate capsulotomy pattern without direct comparison with other techniques. The observed associations between total laser energy and early densitometric changes were weak and exploratory and should not be interpreted as evidence of a dose–response relationship. Finally, glare, straylight, contrast sensitivity, and several potential optical confounders were not quantitatively assessed.

## 5. Conclusions

In this 51-eye retrospective, self-controlled cohort, cruciate-pattern Nd:YAG posterior capsulotomy was associated with small-to-moderate regional increases in corneal backscatter and marked improvement in decimal BCVA over one month. ECD and hexagonality showed no statistically significant changes, while CCT, keratometric parameters, and measured corneal aberrations remained stable. The isolated reduction in CV and the weak correlations between laser energy and early densitometric changes should be interpreted cautiously. These observations are reassuring for the measured short-term outcomes, but do not demonstrate endothelial equivalence or justify changing current capsulotomy technique. Further prospective controlled studies with longer follow-up are warranted specifically to determine whether the regional corneal densitometry changes observed after Nd posterior capsulotomy persist, resolve, or progress over time, and whether these changes are associated with subsequent endothelial alterations or measurable effects on visual quality, including contrast sensitivity and glare.

## Figures and Tables

**Table 1:** Snellen and decimal visual acuity levels with their corresponding logMAR values.

* logMAR	Decimal Visual Acuity	Approximate Snellen Equivalent
−0.3	2.00	20/10
−0.2	1.60	20/12.5
−0.1	1.25	20/16
0.0	1.00	20/20
0.1	0.80	20/25
0.2	0.63	20/32
0.3	0.50	20/40
0.4	0.40	20/50
0.5	0.32	20/63
0.6	0.25	20/80
0.7	0.20	20/100
0.8	0.16	20/125
0.9	0.125	20/160
1.0	0.10	20/200
1.1	0.08	20/250
1.2	0.063	20/320
1.3	0.05	20/400
1.4	0.04	20/500
1.5	0.032	20/630
1.6	0.025	20/800
1.7	0.02	20/1000
1.8	0.016	20/1250
1.9	0.0125	20/1600
2.0	0.01	20/2000

* logMAR: Logarithm of the Minimum Angle of Resolution.

**Table 2:** Demographic, Clinical, and Procedure-Related Characteristics of Patients Undergoing Cruciate-Pattern Nd:YAG Laser Posterior Capsulotomy (*n* = 51).

Variable	Category	Value
Age, y		67.1 ± 8.1/68 (43–79)
Sex, *n* (%)	Male	22 (43.1)
	Female	29 (56.9)
Eye, *n* (%)	Right	25 (49.0)
	Left	26 (51.0)
IOL type, *n* (%)	Foldable	51 (100)
Refraction, *D*		−0.90 ± 1.86/−0.75 (−10 to +3)
Interval from cataract surgery to laser capsulotomy, d		1133.02 ± 1473.98/740 (32–7200)
Pulse energy, mJ		1.02 ± 0.05/1.00 (1.0–1.3)
Total pulse count		120.7 ± 68.8/115 (26–287)
Total laser energy, mJ		119.22 ± 68.13/114 (21–287)
Capsulotomy diameter, mm		4.39 ± 0.51/4.30 (3.30–5.30)

Data are presented as mean ± SD/median (minimum–maximum) for continuous variables and *n* (%) for categorical variables. Abbreviations: *D*, diopters; IOL, intraocular lens; mJ, millijoules; SD, standard deviation.

**Table 3:** Regional and layer-specific corneal densitometry before and after cruciate-pattern Nd:YAG capsulotomy (*n* = 51).

Layer	Zone (mm)	Preoperative	Postoperative Week 1	Postoperative Month 1	Effect Size (Cohen d)			*p* Value
		Mean ± SD	Mean ± SD	Mean ± SD	Pre–W1	Pre–M1	W1–M1	
Anterior	0–2	21.75 ± 2.95 ^b,c^	22.43 ± 2.87 ^a^	23.02 ± 2.60 ^a^	0.23	0.46	0.22	<0.001
	2–6	21.53 ± 3.81 ^b,c^	22.61 ± 3.93 ^a^	22.84 ± 3.43 ^a^	0.28	0.36	0.06	<0.001
	6–10	36.46 ± 11.58 ^b,c^	38.94 ± 12.89 ^a^	38.83 ± 12.52 ^a^	0.20	0.20	0.01	<0.001
	10–12	43.46 ± 14.73	44.74 ± 15.24	44.14 ± 14.56	0.09	0.05	0.04	0.540
	Total	29.87 ± 6.70 ^b,c^	31.26 ± 7.51 ^a^	31.33 ± 6.94 ^a^	0.20	0.21	0.01	0.001
Central	0–2	15.31 ± 1.80 ^c^	15.70 ± 1.75 ^c^	16.21 ± 1.63 ^a,b^	0.22	0.52	0.30	<0.001
	2–6	15.23 ± 2.19 ^b,c^	15.84 ± 2.22 ^a^	16.10 ± 2.00 ^a^	0.28	0.42	0.12	<0.001
	6–10	24.96 ± 6.45 ^b,c^	26.65 ± 7.38 ^a^	26.64 ± 6.93 ^a^	0.24	0.25	0.00	<0.001
	10–12	26.48 ± 5.89	27.39 ± 6.43	27.29 ± 5.19	0.15	0.15	0.02	0.210
	Total	20.21 ± 3.54 ^b,c^	21.17 ± 4.03 ^a^	21.30 ± 3.63 ^a^	0.25	0.30	0.03	<0.001
Posterior	0–2	9.87 ± 1.25	10.03 ± 1.86	10.25 ± 1.48	0.10	0.28	0.13	0.220
	2–6	10.26 ± 1.52	10.59 ± 1.88	10.70 ± 1.69	0.19	0.27	0.06	0.080
	6–10	17.11 ± 3.45 ^b,c^	17.96 ± 4.08 ^a^	18.00 ± 3.85 ^a^	0.23	0.24	0.01	0.007
	10–12	21.03 ± 3.92	21.42 ± 4.79	21.70 ± 3.66	0.09	0.18	0.07	0.370
	Total	14.13 ± 2.14 ^c^	14.61 ± 2.59	14.67 ± 2.40	0.20	0.24	0.02	0.040
Full-thickness	0–2	15.65 ± 1.80 ^c^	16.06 ± 1.91 ^c^	16.50 ± 1.64 ^a,b^	0.22	0.49	0.25	<0.001
	2–6	15.67 ± 2.37 ^b,c^	16.35 ± 2.49 ^a^	16.54 ± 2.20 ^a^	0.28	0.38	0.08	<0.001
	6–10	26.18 ± 7.00 ^b,c^	27.82 ± 7.97 ^a^	26.82 ± 7.59 ^a^	0.22	0.09	0.13	<0.001
	10–12	30.33 ± 7.64	31.23 ± 8.08	31.04 ± 7.10	0.11	0.10	0.03	0.360
	Total	21.40 ± 3.99 ^b,c^	22.35 ± 4.56 ^a^	22.43 ± 4.15 ^a^	0.22	0.25	0.02	0.001

Superscripts indicate significant pairwise differences: *a*, different from the preoperative value; *b*, different from postoperative week 1; *c*, different from postoperative month 1. *p* values were obtained using repeated-measures ANOVA. Post-hoc pairwise comparisons were performed with Bonferroni correction. *p* < 0.05 was considered statistically significant. Corneal densitometry values are expressed in grayscale units (GSU). Abbreviations: *M1*, postoperative month 1; SD, standard deviation; W1, postoperative week 1.

**Table 4:** Visual, Intraocular Pressure, Corneal Endothelial, Keratometric, and Aberrometric Parameters at Baseline, Postoperative Week 1, and Postoperative Month 1, With Effect Sizes (*n* = 51).

Parameter	Preoperative	Postoperative Week 1	Postoperative Month 1	Effect Size (Cohen d)			*p* Value
	Mean ± SD	Mean ± SD	Mean ± SD	Pre–W1	Pre–M1	W1–M1	
**BCVA**	0.32 ± 0.18 ^b,c^	0.71 ± 0.15 ^a,c^	0.81 ± 0.13 ^a,b^	2.35	3.12	0.71	<0.001 *
**IOP**	15.6 ± 2.36	15.0 ± 3.14 ^c^	16.4 ± 2.73 ^b^	0.22	0.31	0.48	0.004 *
**CCT**	527.6 ± 31.5	528.1 ± 31.5	525.8 ± 33.6	0.02	0.06	0.07	0.350 *
**K1**	44.01 ± 1.53	44.01 ± 1.53	44.10 ± 1.58	0.00	0.06	0.06	0.260 *
**K2**	44.78 ± 1.71	44.82 ± 1.68	44.90 ± 1.75	0.02	0.07	0.05	0.170 *
**Kmax**	45.88 ± 1.72	45.94 ± 1.74	46.14 ± 1.80	0.03	0.15	0.11	0.200 *
**CV**	36.4 ± 6.9 ^c^	34.4 ± 6.6	33.3 ± 6.6 ^a^	0.30	0.46	0.17	0.003 *
**Hexagonality**	51.5 ± 13.9	55.0 ± 13.1	51.5 ± 13.5	0.26	0.00	0.26	0.290 *
**Total RMS**	2.14 ± 1.00	2.16 ± 0.85	2.24 ± 1.12	0.02	0.09	0.08	0.510 *
**RMS HOA**	0.74 ± 0.24	0.72 ± 0.24	0.76 ± 0.28	0.08	0.08	0.15	0.360 *
**Spherical aberration**	0.33 ± 0.14	0.30 ± 0.12	0.35 ± 0.15	0.23	0.14	0.37	0.060 *
**Coma 90°**	0.09 (−0.18 to 0.44)	0.11 (−0.21 to 0.36)	0.07 (−0.16 to 0.44)	—	—	—	0.940 †
**Coma 0°**	0.04 (−0.05 to 0.16)	0.01 (−0.09 to 0.14)	0.04 (−0.10 to 0.16)	—	—	—	0.360 †
**ECD**	1694 (1337–1888)	1707 (1427–1941)	1710 (1398–1847)	—	—	—	0.720 †

Data are presented as mean ± standard deviation (SD) for variables analyzed using * repeated-measures ANOVA and as median (Q1–Q3) for variables analyzed using the † Friedman test. When a statistically significant overall time effect was detected, post-hoc pairwise comparisons were performed with Bonferroni correction. Superscripts indicate significant pairwise differences: *a*, different from the preoperative value; *b*, different from postoperative week 1; *c*, different from postoperative month 1.

**Table 5:** Correlation Between Total Nd:YAG Laser Energy and Changes in Corneal Densitometry (*n* = 51).

Layer	Zone (mm)	Pre–W1		Pre–M1		W1–M1	
		r	*p*	r	*p*	r	*p*
Anterior	0–2	0.052	0.715	0.095	0.505	0.063	0.662
	2–6	0.153	0.284	0.126	0.380	−0.025	0.861
	6–10	0.316	**0.024**	0.135	0.346	−0.222	0.118
	10–12	0.021	0.886	−0.110	0.442	−0.139	0.331
	Total	0.205	0.150	0.051	0.721	−0.144	0.313
Central	0–2	−0.095	0.507	0.002	0.991	0.099	0.488
	2–6	0.066	0.648	0.085	0.552	0.028	0.843
	6–10	0.255	0.071	0.116	0.417	−0.178	0.212
	10–12	−0.089	0.532	−0.103	0.471	−0.019	0.895
	Total	0.116	0.418	0.042	0.770	−0.080	0.576
Posterior	0–2	0.174	0.222	0.206	0.147	−0.007	0.962
	2–6	0.221	0.119	0.191	0.179	−0.068	0.636
	6–10	0.295	**0.035**	0.166	0.245	−0.152	0.287
	10–12	−0.038	0.790	0.093	0.516	0.136	0.341
	Total	0.207	0.145	0.169	0.235	−0.049	0.734
Full-thickness	0–2	0.063	0.661	0.109	0.444	0.066	0.645
	2–6	0.176	0.217	0.154	0.279	−0.019	0.897
	6–10	0.292	**0.037**	0.146	0.305	−0.183	0.199
	10–12	−0.022	0.876	−0.071	0.620	−0.060	0.677
	Total	0.194	0.173	0.081	0.573	−0.112	0.436

Note: Pre–W1 = Week 1 − baseline; Pre–M1 = Month 1 − baseline; W1–M1 = Month 1 − Week 1. r, Pearson correlation coefficient. A *p*-value < 0.05 was considered statistically significant.

## Data Availability

The datasets generated and/or analyzed during the current study are available from the corresponding author on reasonable request.

## Abbreviations

The following abbreviations are used in this manuscript:

Abbreviation	Definition
ANOVA	Analysis of variance
BCVA	Best-corrected visual acuity
CCT	Central corneal thickness
CD	Corneal densitometry
CV	Coefficient of variation of endothelial cell area
D	Diopters
ECD	Endothelial cell density
GSU	Grayscale units
HEX	Percentage of hexagonal endothelial cells
HOA	Higher-order aberrations
IOL	Intraocular lens
IOP	Intraocular pressure
K1	Flat keratometry
K2	Steep keratometry
Kmax	Maximum keratometry
M1	Postoperative month 1
Nd:YAG	Neodymium:yttrium–aluminum–garnet
PC	Posterior capsulotomy
PCO	Posterior capsule opacification
Q1	First quartile
Q3	Third quartile
RMS	Root mean square
RMS HOA	Root mean square of higher-order aberrations
SD	Standard deviation
W1	Postoperative week 1
Units and Symbols	
%	Percent
°	Degrees
cells/mm^2^	Cells per square millimeter
d	Days
Cohen’s d	Standardized effect size
mJ	Millijoules
mm	Millimeters
mm Hg	Millimeters of mercury
μm	Micrometers
*n*	Number of observations or patients

## References

[1] Lindholm J.M., Laine I., Tuuminen R. (2019). Five-Year Cumulative Incidence and Risk Factors of Nd:YAG Capsulotomy in 10 044 Hydrophobic Acrylic 1-Piece and 3-Piece Intraocular Lenses. Am. J. Ophthalmol..

[2] Mutaf C., Reyhan A.H., Bulut M., Yüksekyayla F. (2025). Ultra-Short-Term Corneal Changes to Nd:YAG Laser Capsulotomy: Energy-Dependent Changes Assessed by Specular Microscopy and Topographic Analysis. Diagnostics.

[3] Samir A.M., Haroun H.E., El Ghonemy H.M.E., El-Nor R.M.A. (2022). Effect of Nd:YAG Laser Capsulotomy on Corneal Endothelium. Egypt. J. Med. Res..

[4] Borkenstein A.F., Borkenstein E.M. (2024). Precise Posterior Nd:YAG Capsulotomy without Creating Defects Is Key for Patients’ Quality of Vision. Clin. Ophthalmol..

[5] Meyer K.T., Pettit T.H., Straatsma B.R. (1984). Corneal Endothelial Damage with Neodymium:YAG Laser. Ophthalmology.

[6] Vaikoussis E., Bisogiannis Z., Margaritis L. (1993). Corneal Endothelial Damage after Nd:YAG Laser Anterior Capsulotomy: An Experimental Study on Rabbits. Doc. Ophthalmol..

[7] Arizcuren A.R., Remon L., Consejo A. (2025). Influence of Brightness Artefacts on Corneal Densitometry. Ophthalmic Physiol. Opt..

[8] Oncul H., Yildirim Y., Caglayan M., Alakus M.F. (2021). The Effect of Chalazion Excision on Corneal Aberrometric and Densitometric Values. Beyoglu Eye J..

[9] Elgin U., Şen E., Şimşek M., Budakoğlu Ö., Yıldırım D., Yeşilyaprak N. (2021). The Effect of Laser Peripheral Iridotomy on Corneal Transparency in Patients with Acute Primary Angle Closure Glaucoma. J. Glaucoma-Cataract..

[10] Yıldırım Y. (2026). Localized Peripheral Corneal Densitometry Changes at 1 Month after Laser Posterior Capsulotomy. Korean J. Ophthalmol..

[11] Akmaz B., Akay F. (2018). Evaluation of the Anterior Segment Parameters after Nd:YAG Laser Capsulotomy: Effect of the Design of Intraocular Lens Haptic. Pak. J. Med. Sci..

[12] Kara N., Evcimen Y., Kirik F., Agachan A., Yigit F.U. (2014). Comparison of two laser capsulotomy techniques: Cruciate versus circular. Semin. Ophthalmol..

[13] Kim J.S., Choi J.Y., Kwon J.W., Wee W.R., Han Y.K. (2018). Comparison of two Nd:YAG laser posterior capsulotomy: Cruciate pattern vs circular pattern with vitreous strand cutting. Int. J. Ophthalmol..

[14] Li J., Yu Z., Song H. (2020). The Effect of Capsulotomy Shape on Intraocular Light-Scattering after Nd:YAG Laser Capsulotomy. J. Ophthalmol..

[15] Cohen J. (1988). Statistical Power Analysis for the Behavioral Sciences.

[16] Sullivan G.M., Feinn R. (2012). Using effect size—Or why the P value is not enough. J. Grad. Med. Educ..

[17] Zhang J., Hu H., Nie D., Sun L., He W., Liu X. (2023). Age-Related Changes in Scheimpflug Corneal Densitometry and Their Correlations with Corneal Topographic Measurements in a Healthy Chinese Population. Quant. Imaging Med. Surg..

[18] Keleş A., Karaman S. (2021). Nd:YAG Lazer Kapsülotominin Göz İçi Basınç ve Kornea Endoteli Üzerine Erken Dönemde Etkisi. Akd. Med. J..

[19] Özen O., İnal Özen M., Örnek F. (2026). Comparison of Corneal Endothelial and Anterior Segment Changes after Nd:YAG Laser Capsulotomy in Eyes with and without Pseudoexfoliation Syndrome. Semin. Ophthalmol..

[20] Öztürk Y., Göktaş E., Çallı Ü., Yaprak D.Ç., Oral Y., Penbe A. (2019). Corneal Endothelial Changes Following Nd:YAG Laser Capsulotomy in Patients with Dense Posterior Capsular Opacification. Turk. Klin. J. Ophthalmol..

[21] Chen H.C., Lee C.Y., Liu C.F., Hsueh Y.-J., Meir Y.-J.J., Cheng C.-M., Wu W.-C. (2022). Corneal Endothelial Changes Following Early Capsulotomy Using Neodymium:Yttrium-Aluminum-Garnet Laser. Diagnostics.

[22] Ono T., Iida M., Sakisaka T., Minami K., Miyata K. (2018). Effect of Laser Peripheral Iridotomy Using Argon and Neodymium-YAG Lasers on Corneal Endothelial Cell Density: 7-Year Longitudinal Evaluation. Jpn. J. Ophthalmol..

[23] Ghani S.I., Zunaina E. (2021). Effect of 532 nm Argon Laser Pan Retinal Photocoagulation on Corneal Thickness and Corneal Endothelial Cell Parameters among Proliferative Diabetic Retinopathy Patients. J. Diabetes Metab. Disord..

[24] Neklawi M.F.A., Selim K.M., Kazzaz A.-R.R.Z.E. (2024). Corneal Endothelial Changes after Panretinal Photocoagulation in Diabetic Patients. AL-Azhar Int. Med. J..

[25] Paranjpe R., Gandhi S., Bhavsar D., Goyal K., Agrawal T., Goli K.B. (2024). Evaluation of Intraocular Pressure, Refraction, Anterior Chamber Depth, Macular Thickness, and Specular Microscopy Post-Neodymium-Doped Yttrium-Aluminum-Garnet Laser in Patients with Posterior Capsular Opacification. Cureus.

[26] Alakuş M.F., Kozan B.D., Çağlayan M., Öncül H., Kılıç J. (2026). Tiroid Hormon Seviyesinin Korneanın Topografik Dansitometrik ve Aberrometrik Değerleri Üzerindeki Etkileri. Ege Tıp Derg..

[27] Coskun C., Celik G., Zeki Fikret C., Çomçalı S., Kemer Ö.E. (2024). Evaluation of Corneal Densitometry Values with Pentacam in Cases of Ocular Hypertension and Pseudoexfoliative Glaucoma. Photodiagnosis Photodyn. Ther..

[28] Bozali E., Yeter D.Y., Tosun M., Apa A.S. (2024). Assessment by the Scheimpflug Imaging System of Corneal Clarity and Anterior Segment Properties in Rosacea Patients. An. Bras. Dermatol..

[29] Alberdi T., Mendicute J., Bascaran L., Ruiz-Ederra J. (2018). Anterior and Posterior Capsule Densitometry Levels after Femtosecond Laser-Assisted Cataract Surgery. Int. J. Ophthalmol..

